# The Impact of an Authentic Sports Leadership Program for Coach

**DOI:** 10.3389/fpsyg.2021.701134

**Published:** 2021-06-21

**Authors:** Diego Soto Garcia, Juan Antonio García Herrero, Rodrigo Jesús Carcedo, Mario Sánchez García

**Affiliations:** ^1^Department of Physical and Sports Education, Universidad de León, León, Spain; ^2^Department of Didactics of Musical, Plastic and Body Expression, University of Salamanca, Salamanca, Spain; ^3^Department of Developmental and Educational Psychology, University of Salamanca, Salamanca, Spain; ^4^Education Faculty, Pontifical University of Salamanca, Salamanca, Spain

**Keywords:** sport coaching, identity, influence, self-awareness, football, handball

## Abstract

**Purpose:** This paper studies the effects of authentic sports leadership training on coaches' self-perception of their own authentic leadership, perceived justice, competence, overall self-efficacy, and collective efficacy. Additionally, players' perceptions of their coaches with respect to their authentic leadership, perceived justice, competence, collective efficacy, satisfaction with the coach, and support for basic psychological needs were analyzed.

**Design:** Twenty-five football and handball coaches were randomly assigned to two groups. Fifteen coaches made up the experimental group that carried out the training leadership program, while 10 coaches made up the control group, carrying out no training whatsoever. A total of 248 football and handball players participated in this study; 136 were led by coaches who participated in the training program, and 112 by coaches who did not participate in the program.

**Results:** The results of this study indicate that coaches' self-perception is positively influenced after having received training in the variables of authentic leadership, perceived justice, competence, overall self-efficacy, and collective efficacy. Players whose coaches were part of the program perceive them as being more competent as coaches.

**Conclusions:** The effects of an authentic sports leadership training program are effective for coaches and players alike.

## Introduction

“He was born to be a leader” or “she is a natural leader” are frequent expressions in different contexts where the figure of a leader is present. In the realm of sports, numerous studies attribute a fundamental aspect of sportsman to the coach (Adie et al., 2012; Balaguer et al., 2012), making him or her one of the most significant actors in terms of team sports performance (Myers et al., 2011). But can good leadership be learned, and is it possible to train coaches in these skills? Northouse (2018), maintains that there is a whole series of beliefs surrounding leadership, which opens the door to the ongoing debate of whether leadership is innate, or conversely, a skill that can be developed by coaches and leaders. As in other social activities, in sports it is assumed that leadership skills can be acquired through training programs (Duda, 2013). Hence, in recent decades there has been a growing interest in the study of leadership models and how to apply them. In many cases, these models have been oriented toward interactions and context (Epitropaki et al., 2017), thus, the coach's influence and how it is perceived by athletes has become increasingly central to various conceptualizations and analyses (Steffens et al., 2016; Epitropaki et al., 2017).

In recent years, there has been a growing interest in studying the relationship between effective leadership and the ethical behavior of the leader (Kaptein, 2019; Gamarra and Girotto, 2021). For this reason, models oriented toward the ethical dimension of team leadership have recently emerged, such as what has been termed “authentic leadership,” (Avolio and Gardner, 2005; Gardner et al., 2005a; Moriano et al., 2011). Walumbwa et al. (2008) which posit that authentic leadership comprises four related dimensions: self-awareness, internalized moral perspective, relational transparency, and balanced processing.

Self-awareness refers to the extent which a leader possesses accurate self-knowledge and uses that knowledge to demonstrate he or she is cognizant of their impact on others. Internalized moral perspective pertains to the degree in which a leader's behavior is directed by and is congruent with their personal values and moral standards. It represents a form of self-regulation which allows leaders to engage in ethical conduct even against external pressures. Relational transparency is a leader's presentation of their true thoughts and emotions in an open and transparent manner (vs. being fake or manipulative), while balanced processing is evident when a leader objectively considers and analyzes all relevant information before making decisions (Walumbwa et al., 2008).

Various contextually different studies have shown that authentic leadership is related to the well-being of the leader (Baron, 2012; Gatling and Harrah, 2014; Weiss et al., 2018), additionally improving followers' behavior, satisfaction, and performance (Walumbwa et al., 2008; Caza et al., 2010). In the realm of sports, it has been associated with higher levels of competence and perceived justice on the part of the coach (González-Ponce, 2018), as well as increased satisfaction, enjoyment and cohesion among the team (Bandura and Kavussanu, 2018), as well as greater team cohesion; a relationship mediated by trust and sacrifice (Bandura et al., 2019). A study by McDowell et al. (2018) involving basketball players, the positive team climate presented a mediating effect between authentic leadership and psychological capital and player commitment.

Two of the most influential approaches to sports leadership have been: Leadership Behaviors Model of Sport Leadership (Smoll et al., 1978; Smoll and Smith, 1989) and Multidimensional Model of Sport Leadership (Chelladurai, 1978, 1990, 2007). There have been several programs aimed at training coaches in sports leadership skills which predominantly follow these approaches. One of the pioneers and most widely used is Coach Effectiveness Training (CET), by Smith et al. (1979), which aims to facilitate positive behavior on the part of coaches; likewise, to improve interactions with their athletes. Several studies have supported the usefulness of CET, resulting in more effective coach behaviors and better interactions (Sousa et al., 2008). Based on CET, the Mastery Approach to Coaching (MAC) (Smith et al., 2007; Smoll and Smith, 2009) emphasizes the importance of a task-oriented climate as generated by the coach rather than results-oriented. In this line, the MAC also recommends that coaches avoid post-error punishment and punitive technical instruction (Smoll and Smith, 2010; Vella and Perlman, 2014) in order to facilitate positive athletic behavior and promote a task-oriented climate.

The Transformational Leadership Approach to Coaching (TLAC) is another proposed approach to developing leadership skills. In essence, transformational leaders promote autonomous actions, pursuing the personal growth of athletes, as well as task cohesion, the satisfaction of needs, and intrinsic motivation (Vella and Perlman, 2014). In youth sport contexts, the TLAC has been implemented with the aim of facilitating positive developmental outcomes among athletes (Vella et al., 2012, 2013) and has been employed to facilitate high performance among Olympic athletes as well (Din and Paskevich, 2013).

The convenience of this study is due to the influence that the coach has on the athletes, and also, putting in value the positive effects that authentic leadership has on athletes (Bandura and Kavussanu, 2018), it could be considered transcendent to seek ways to develop this style of leadership and observe the effects on different variables associated with the abilities to lead by the coach and how it affects the athletes.

The objective of this study was to investigate the effect of an authentic sports leadership training program on several variables affecting both the coaches participating in the program and their players. The efficacy of this intervention was tested by both coaches and their players using a before and after study design. Authentic coach leadership, perceived justice, coach competence, and collective efficacy was evaluated by the coaches. The same variables (excluding coach self-efficacy) along with the satisfaction with the coach and perceived need satisfaction was evaluated by the players. As a result, two main hypotheses were developed:

1. From the perspective of the coaches, it is expected that those coaches who received the training program (experimental group) will show a significant increase in the evaluation of their levels of authentic coach leadership, perceived justice, coach competence (total competence, motivation, decision-making, teaching and instruction of skills, and character influence), coach self-efficacy, and collective efficacy after the intervention in comparison with before, whereas no changes will be found in those coaches who did not receive the intervention (control group).
2. From the evaluation of the players, it is expected that those players whose coaches have received the training program (experimental group) will show an increase in their perception of their coach's authentic leadership, perceived justice, coach competence (total competence, motivating, decision-making, teaching and instruction of skills, and character influence), collective efficacy, satisfaction with the coach, and coach support of their basic psychological needs (competence, autonomy, and relatedness).

## Method

### Participants

The training course was offered to all students of the National Degree of Higher Sports Technicians from Spain. A total of 25 coaches agreed to participate, all male, they were randomized; 15 coaches made up the experimental group and 10 coaches made up the control group. The average age of the participants in the experimental group was 36.31(*SD* = 8.07), whereas the average age among the control group was 31.72 (*SD* = 5.45). The experimental group had an average of 10.53 years of experience as head coach (*SD* = 6.54), with the control group having had 8.50 average years of experience (*SD* = 2.99).

A total of 248 football and handball players participated in this study, 178 being male, and 70 female; 136 were led by coaches from the experimental group who had received leadership training, and 112 were led by coaches from the control group. The average age of the participants was 17.84 years (*SD* = 4.44). The athletes had an average of 10.45 years of experience (*SD* = 4.83) participating in the sport being played. Finally, all the coaches and the players trained more than 8 h per week and compete in a regional category.

### Variables and Instruments

#### Authentic Coach Leadership

This variable was evaluated according to the Spanish adaptation validated by Moriano et al. (2011) of the Authentic Leadership Questionnaire (ALQ) (Walumbwa et al., 2008). This instrument consists of 12 items. The answer format is a Likert scale from 1 (never) to 7 (always). Following Gadermann et al. (2012) recommendations, internal consistency reliability was calculated using Omega coefficients. Good levels were found for the coaches (before the intervention: ω = 0.91; after the intervention: ω = 0.91) and the players (before: ω = 0.93; after: ω = 0.92).

### Perceived Justice

This variable was evaluated using an adaptation of Colquitt's Organizational Justice scale (2001) by García-Calvo et al. (2014a) of which a modification was made in order to measure perceived justice within a sport-specific framework. The instrument consists of 12 items and the response format is a Likert scale from 1 (never) to 7 (always). Internal consistency reliability showed good levels for the coaches (before: ω = 0.88; after: ω = 0.85) and the players (before: ω = 0.91; after: ω = 0.91).

### Coach Competence

This variable was evaluated by means of Athletes Perceptions of Coaching Competency Scale II-High School Teams (APCCS II-HST; Myers et al., 2010) validated in Spanish (González-Ponce et al., 2017). There are 15 items in this instrument and four subscales: competence to motivate, decision-making, teaching and instruction of skills, and influence on the player. The response format is a Likert scale from 1 (incompetence) to 7 (full competence). Good levels of internal consistency reliability were found for both coaches (motivate: before: ω = 0.63, after: ω = 0.85; decision-making: ω = 0.76, after: ω = 0.77; teach and instruct: ω = 0.89, after: ω = 0.88; influence: ω = 0.77, after: ω = 0.80), and players (motivate: before: ω = 0.78, after: ω = 0.78; decision-making: ω = 0.79, after: ω = 0.84; teach and instruct: ω = 0.79, after: ω = 0.79; influence: ω = 0.70, after: ω = 0.72).

### Coach Self-Efficacy

This variable was evaluated using the Scale of Self Efficacy of Baessler and Schwarzer (1996) based on the Spanish version developed by Sanjuán et al. (2000). This instrument consists of 10 items. The response format is a Likert scale from 1 (strongly disagree) to 4 (strongly agree). Only the coaches gave their responses on this scale. Internal consistency reliability also showed good levels in this case (before: ω = 0.84; after: ω = 0.92).

### Collective Efficacy

This variable was evaluated using a scale developed by Leo et al. (2010). This instrument consists of 6 items. The response format is a Likert scale from 1 (poor) to 5 (excellent). Good levels of internal consistency reliability were found for both coaches (before: ω = 0.83; after: ω = 0.63) and players (before: ω = 0.71; after: ω = 0.75).

### Satisfaction With the Coach

This variable was evaluated using an adaptation of the Myers et al. (2011) in its Spanish version. This instrument consists of 3 items and represents a reduced version adapted to the Spanish context (González-Ponce et al., 2015). The response format is a Likert scale from 1 (very little) to 5 (a lot). This scale was only responded to by the players. Internal consistency reliability also showed good levels in this case (before: ω = 0.80; after: ω = 0.83).

### Perception of Support for Basic Psychological Needs

This variable was evaluated using the Psychological Needs Support Questionnaire (CANPB; Sánchez-Oliva et al., 2013) adapted to the sports context. This instrument consists of 12 items distributed across three dimensions: autonomy, competence, and need for relatedness. The response format is a Likert scale from 1 (totally disagree) to 5 (totally agree). Only players responded to this scale. Again, good levels of internal consistency were observed in the three subscales (autonomy: before: ω = 0.81, after: ω = 0.76; competence: before: ω = 0.80, after: ω = 0.77; relatedness: before: ω = 0.87, after: ω = 0.76).

### Procedure

This study was conducted applying a before/after design with an experimental group and a control group. Prior to the intervention, initial questionnaires were administered to all coaches and teams. For the coaches, the questionnaires were administrated outside the sporting context in which they regularly perform their duties as coaches. Conversely, the athletes were taken to the facility where they habitually train and explained the guidelines for completing the questionnaires by the researcher. Any related questions were also addressed at this time. The questionnaires were administered to the athletes collectively, with all members of the team present. The researcher remained on-site and available to the participants at all times should any questions arise. In both cases, the time used by both coaches and athletes was ~25–30 min. For coaches and players in both groups, there was a 7-week period between the first and second administration of the questionnaires.

This study respected the norms of the Declaration of Helsinki. Additionally, the parents of the minors were informed and all participants signed consent forms.

### Description of the Program

Authentic leadership seeks to move away from unethical actions, respecting and displaying values that inspire others to join in that direction. According to George (2015, p. 1), “authentic leaders are people committed to meeting the needs of the interest groups they serve, displaying values and self-discipline that inspire others.” Our training program was oriented toward this behavior, considering the four dimensions proposed in the Authentic Leadership Questionnaire (ALQ) by Walumbwa et al. (2008): transparency in relationships, internalized morality, balanced processing and self-awareness. The duration of the training program was 15 h divided into four sessions developed within three consecutive days. The structure and contents of the training program took into account the “model for the development of the moral component of authentic leadership” proposed by May et al. (2003). This model consists of three key elements: authenticity in decision-making, authentic behavior, and development of authentic leadership, each of which was present in the training program, especially in sessions 2, 3, and 4. Similarly, in the implementation of the program, “the integral model for the development of the authentic leader and his followers” proposed by Gardner et al. (2005b) was considered. The approach of this model is oriented toward the leader's self-awareness (identity, values and motivations) and self-regulation (balanced processing and transparency in relationships); in particular the contributions of this model were used in sessions 1, 2, and 3 as detailed below. The following table describes the contents and timing of the program.

### Program Sessions

Session 1 (4 h). Transparency in relationships. In this session, the program was presented and the relationships that exist in sports groups were developed through inquiry tasks. Tasks were carried out in small groups where the meaning and purpose of relationships and the role of the coach among them were discussed. Communication skills and active listening were developed with the coaches, linking these components to sports environments. Through group dynamics these skills were put into practice, seeking that the coaches could show themselves as they are, without fear of judgments or external evaluations.

Session 2 (4 h). Internalized morality. This session was divided into two main blocks, the first dedicated to the work of the coach's self-awareness (content that was also developed in session 4). Through role play and reflective tasks, the coaches identified their personal values that made sense in leadership with their teams. How to approach ethical behavior and how to maintain authenticity in their conduct from a moral point of view was shared with the coaches through reflective tasks. The second block of this session was aimed at connecting the identity of the coach with the identity and values of his teams. Through role-playing games, everyday situations were put into practice among the teams, the coach being required to recognize frequently recurring moral dilemmas. The worked out was in a way that respected personal values and the identity of the team.

Session 3 (4 h). Balanced processing. Ethical decision-making seeks to objectively examine the variables involved in accepting personal responsibility for actions, results, and mistakes. In this session, the coaches focused on the ethics of decisions made in the teams, seeking to align performance with the value system seen in the previous session. In the same way, information channels and sources of feedback available to the coach were identified (even if they were contrary to his positions). Group dynamics, self-reflective tasks and role-playing games were used where coaches were proposed to commonly experience ethical conflicts with sports teams. Decisions related to authentic leadership were identified as those which respected the relationship between the personal values of the coach and team groups.

Session 4 (3 h). Self-awareness. This session was directly linked to session 2, establishing the identity of the coaches, thus strengthening their values and principles. Similarly, through research tasks, coaches identified the professional areas in which they were most and least effective. In small groups they worked to expose resources and procedures that would allow them to pinpoint the least effective areas. Finally, through group dynamics, coaches shared program highlights and learning reported (see Table 1).

**Table 1:** Programming, themes, and contents of the authentic leadership program.

**Session/duration**	**Theme**	**Content**
1/4 h	Relational transparency	Team relationships Communication and listening skills
2/4 h	Internalized morality	Identification of personal and group values Relationship of team identity with group values Awareness and shared responsibility
3/4 h	Balanced processing	Ethics of decision-making Relationship between personal and group decisions and values Respect for the values and identity of the team in decision-making
4/3 h	Self-awareness	Personal effectiveness analysis Resources for managing effectivity and learning reported

### Statistical Analysis

In order to study the program's effectiveness, an ANOVA of repeated measurements was conducted with an intra-subject factor (time of measure: before and after the intervention) and an inter-subject factor (experimental and control groups). *Post-hoc* tests of multiple comparisons with the Bonferroni adjustment were also performed in order to analyze significant interactions. In this way, differences between pre- and post-intervention dependent variables within the experimental and control groups, and differences among the same dependent variables between the experimental and control groups at the time of pre- and post-intervention evaluation could be studied. For all analyses, a significance of 0.05 was established for α.

Finally, it should be mentioned that Omega's alpha was used to evaluate the reliability of the scales used. All analyses were carried out with the SPSS 24.0 statistical package (IBM, Armonk, NY, USA).

## Results

### Coaches

Below are the most relevant results obtained in relation to each of the variables investigated. To evaluate the effectiveness of the program, a series of repeated measurement ANOVAs were performed with an inter-subject factor (experimental vs. control groups) and an intra-subject factor (period of evaluation).

The interaction period of measurement^*^group was statistically significant (see **Table 3**) for the variables authentic leadership [Wilks' λ = 0.643, *F*_(1,22)_ = 12.21, *p* = 0.002, $\eta_{\text{p}}^{2}$ = 0.357], perceived justice [Wilks' λ = 0.724, *F*_(1,22)_ = 8.39, *p* = 0.008, $\eta_{\text{p}}^{2}$ = 0.276], ability to teach and instruct skills to players [Wilks' λ = 0.796, *F*_(1,23)_ = 5.89, *p* = 0.023, $\eta_{\text{p}}^{2}$ = 0.204], general self-efficacy [Wilks' λ = 0.747, *F*_(1,22)_ = 7.45, *p* = 0.012, $\eta_{\text{p}}^{2}$ = 0.253] and collective efficacy [Wilks' λ = 0.773, *F*_(1,23)_ = 6.74, *p* = 0.016, $\eta_{\text{p}}^{2}$ = 0.227].

To interpret these interactions, a series of *post-hoc* tests of multiple comparisons with the Bonferroni adjustment was performed, revealing a significant increase in the levels of authentic leadership (*p* = 0.008), perceived justice (*p* = 0.004), general self-efficacy (*p* = 0.005) and collective efficacy (*p* = 0.002) after the intervention in the experimental group, while no change was found in the control group in these variables. In fact, a significant decrease in this group was observed in the case of authentic leadership (*p* = 0.048). A statistical trend was also found of a marked increase in the ability to teach and instruct skills to players in the post-intervention evaluation in the experimental group (*p* = 0.061), while no variation was observed in the control group.

**Figure 1:**
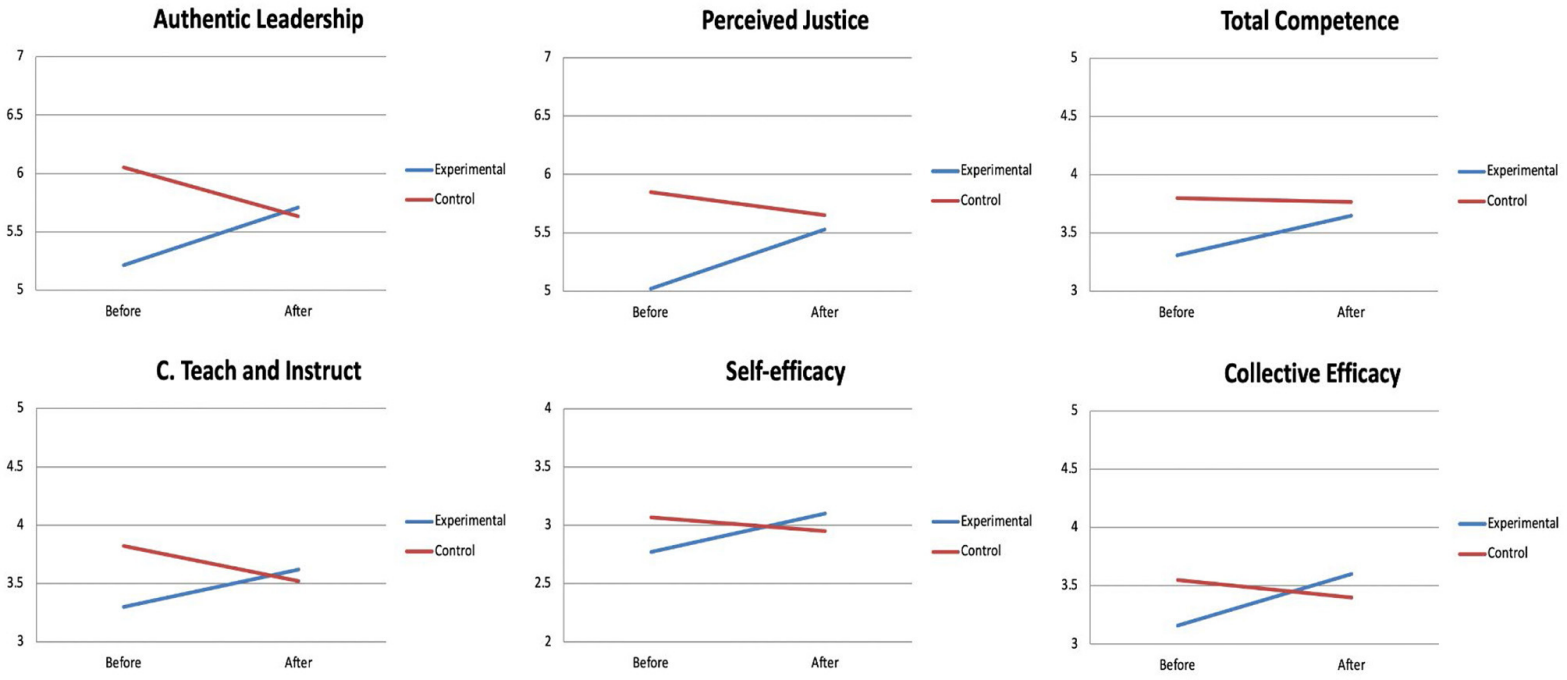
Significant interactions between the time of measure (before vs. after the intervention) and the group (experimental vs. control) for the coaches.

The interaction period of measurement^*^group was not statistically significant for the coach's overall competence variable although a clear statistical trend was observed [*F*_(1,23)_ = 4.00, *p* = 0.057, $\eta_{\text{p}}^{2}$ = 0.148]. When a series of *post-hoc* tests was carried out in accordance with Bonferroni's adjustment, a significant increase in the coaches' competence levels was observed within the experimental group (*p* = 0.007), with no change observed in respect to the control group (see Table 2, and Figure 1).

**Table 2:** Means and standard deviations of the coaches in the experimental and control groups before and after the intervention, and interaction time of measure*group.

	**Before**		**After**		**Interactions**		**Significant interactions**	
	**Exp. group**	**Control group**	**Exp. group**	**Control group**	Time of measure*group		**Exp. group**	**Control group**
	***M***	***M***	***M***	***M***	***p***	**$\eta_{\text{p}}^{2}$**		
	***(SD)***	***(SD)***	***(SD)***	***(SD)***				
Authentic leadership	5.21 (0.66)	6.05 (0.34)	5.71 (0.71)	5.63 (0.60)	0.002	0.357	B < A	B > A
Perceived justice	5.02 (0.61)	5.85 (0.31)	5.53 (0.59)	5.65 (0.57)	0.008	0.276	B < A	B = A
Total competence	3.31 (0.48)	3.80 (0.82)	3.65 (0.44)	3.77 (0.51)	0.057	0.148	B < A	B = A
C. Motivation	3.13 (0.65)	3.80 (0.59)	3.51 (0.71)	3.93 (0.70)	0.340	0.040		
C. Decision-making	3.20 (0.54)	3.50 (0.59)	3.42 (0.53)	3.57 (0.58)	0.413	0.029		
C. Teach and instruct	3.30 (0.64)	3.82 (0.31)	3.62 (0.64)	3.52 (0.45)	0.023	0.204	B < A	B = A
C. Character influence	3.53 (0.74)	4.10 (0.38)	4.08 (0.71)	4.16 (0.65)	0.230	0.062		
Self-efficacy	2.77 (0.34)	3.07 (0.39)	3.10 (0.39)	2.95 (0.50)	0.012	0.253	B < A	B = A
Collective efficacy	3.16 (0.42)	3.55 (0.55)	3.60 (0.52)	3.40 (0.44)	0.016	0.227	B < A	B = A

*B, Before; A, After*.

In addition, no major effect was identified for the period of measurement, however, there was a significant effect for the cluster related to competence to motivate [*F*_(1,23)_ = 5.03, *p* = 0.035, $\eta_{\text{p}}^{2}$ = 0.180] and that of perceived justice [*F*_(1,22)_ = 6.07, *p* = 0.022, $\eta_{\text{p}}^{2}$ = 0.216]. In both cases, the control group (motivational competence: *M* = 3.87; perceived justice: *M* = 5.75) scored higher than the experimental group (motivational competence: *M* = 3.32; perceived justice: *M* = 5.28).

### Players

The most relevant results obtained in relation to each of the variables investigated appear below. To evaluate the effectiveness of the program, a series of repeated measurement ANOVAs was performed with an inter-subject factor (experimental vs. control group) and an intra-subject factor (period of evaluation).

The interaction period of measurement^*^ group was statistically significant (see Table 3) for the variables: coach's overall competence [Wilks' λ = 0.949, *F*_(1,80)_ = 9.58, *p* = 0.002, $\eta_{\text{p}}^{2}$ = 0.051], coach's ability to motivate [Wilks' λ =0.960, *F*_(1,80)_ = 7.43, *p* = 0.007, $\eta_{\text{p}}^{2}$ = 0.040], decision-making ability [Wilks' λ =0.975, *F*_(1,80)_ = 4.55, *p* = 0.034, $\eta_{\text{p}}^{2}$ = 0.025] and ability to positively influence the player's attitude [Wilks' λ =0.965, *F*_(1,80)_ = 6.48, *p* = 0.012, $\eta_{\text{p}}^{2}$ = 0.035].

**Table 3:** Means and standard deviations of the players in the experimental and control groups before and after the intervention and interactions between time of measure*group.

	**Before**		**After**		**Interactions**		**Significant interactions**	
	**Exp. group**	**Control group**	**Exp. group**	**Control group**	Time of measure*group		**Exp. group**	**Control group**
	***M***	***M***	***M***	***M***	***p***	**$\eta_{\text{p}}^{2}$**		
	***(SD)***	***(SD)***	***(SD)***	***(SD)***				
Authentic leadership	5.35 (0.95)	5.43 (1.02)	5.42 (0.83)	5.27 (0.90)	0.057	0	B = A	B > A
Perceived justice	5.50 (0.83)	5.31 (1.04)	5.42 (0.84)	5.22 (0.94)	0.909	0		
Total competence	3.81 (0.59)	3.93 (0.55)	3.88 (0.56)	3.79 (0.60)	0.002	0.051	B = A	B > A
C. Motivation	3.86 (0.74)	4.02 (0.60)	3.94 (0.66)	3.83 (0.65)	0.007	0	B = A	B > A
C. Decision-making	3.52 (0.54)	3.64 (0.59)	3.66 (0.53)	3.57 (0.58)	0.034	0.025	B < A	B = A
C. Teach and instruct	3.87 (0.69)	3.99 (0.66)	3.85 (0.65)	3.83 (0.68)	0.130	0.013		
C. Character Influence	4.08 (0.68)	4.13 (0.70)	4.16 (0.60)	3.96 (0.70)	0.012	0.035	B = A	B > A
Collective efficacy	3.80 (0.66)	3.69 (0.61)	3.81 (0.69)	3.82 (0.50)	0.179	0.010		
Satisfaction with the coach	4.25 (0.61)	4.16 (0.77)	4.32 (0.65)	4.12 (0.89)	0.286	0.006		
PNS autonomy	3.38 (0.73)	3.33 (0.89)	3.53 (0.79)	3.48 (0.77)	0.995	0		
PNS Competence	4.18 (0.55)	4.15 (0.73)	4.12 (0.64)	4.06 (0.64)	0.786	0		
PNS relatedness	4.25 (0.60)	4.21 (0.69)	4.25 (0.59)	4.19 (0.67)	0.891	0		

*B, Before; A, After*.

To interpret these significant interactions, a series of *post-hoc* tests of multiple comparisons was carried out in accordance with the Bonferroni adjustment. First, a significant increase in the coach's decision-making ability (*p* = 0.027) was found in the experimental group, while no change was observed in the control group. A significant decrease in the coach's overall competence (*p* = 0.007), the coach's ability to motivate *(p* = 0.012) and to positively influence the player's attitude (*p* = 0.023) was observed in the control group after the intervention, while the experimental group maintained the same levels as prior to the intervention.

The interaction period of measurement^*^ group was not statistically significant for authentic leadership, although a clear statistical trend was observed [*F*_(1,180)_ = 3.68, *p* = 0.057, $\eta_{\text{p}}^{2}$ = 0.020]. The multiple comparison tests post Bonferroni adjustment only detected a statistical trend indicating a decrease in the control group between before and after the intervention (*p* = 0.079), while the experimental group remained the same.

**Figure 2:**
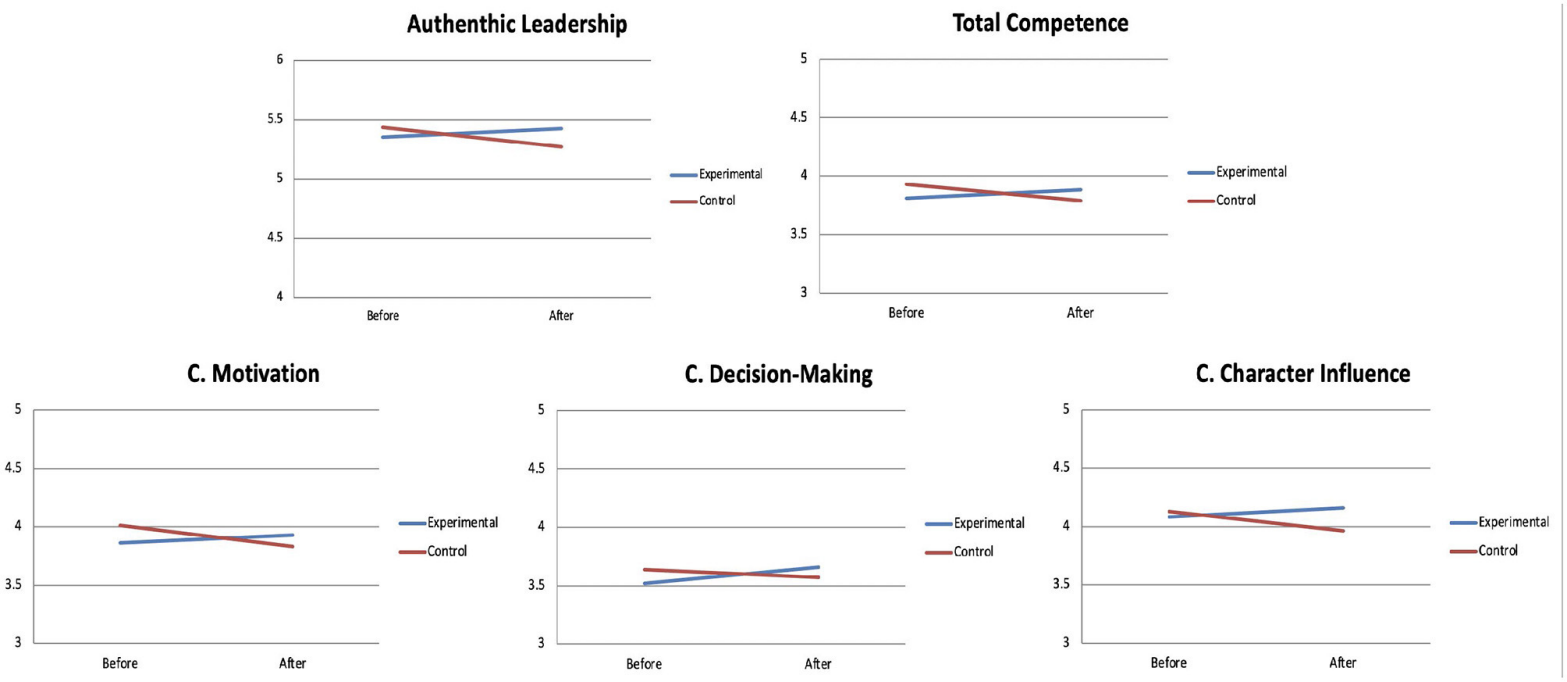
Significant interactions between the time of measure (before vs. after the intervention) and the group (experimental vs. control) for the players.

Finally, it is noteworthy that no major effect was identified for the group except in the case of the period of measurement relating to the need for autonomy [*F*_(1,180)_ = 5.78, *p* = 0.017, $\eta_{\text{p}}^{2}$ = 0.031] and competence [*F*_(1,180)_ = 4.07, *p* = 0.045, $\eta_{\text{p}}^{2}$ = 0.022]. In both cases, higher levels were obtained after the intervention (autonomy: *M* = 3.51; competence: *M* = 4.17) compared to before (autonomy: *M* = 3.36; competence: *M* = 4.09) (see Table 3, and Figure 2).

## Discussion

### Coaches

As results obtained show, the coaches' perception of their own authentic leadership increased significantly for the experimental group, taking into account the before and after measurement period. In the control group, however, self-perception of their own authentic leadership showed a significant decrease. According to the results found in the two groups of coach, the training program had a positive effect on the perception of their leadership style in the experimental group. Results similar to other training programs related to authentic leadership developed in non-sports contexts (Baron, 2012; Fusco et al., 2016; Frasier, 2019).

Paying attention to the relational transparency dimension and the contents developed within the program, it could be considered that communication has an influence on the development of this dimension. Cranmer et al. (2020) relates the coach‘s communication skills to team cohesion and in turn, various studies (Houchin, 2011; González-Ponce, 2018) have revealed positive and significant relationships between authentic leadership and cohesion.

Regarding the development of the internalized moral dimension, role-playing games and reflective tasks that included ethical questioning were used in order to achieve a shared identity that is reflecting the ability of sports leaders to mobilize the efforts of athletes (Slater et al., 2019). The coach's self-perception following the analysis of information related to decision-making and balanced processing could be affected by the contents developed in the authentic leadership program. Since the coach's competence is one of the most determining variables in the performance of sports teams (Myers et al., 2011), it seems essential to acquire and develop the coach's self-reflection on their abilities, thus allowing them to evaluate their impact on others.

The significant increase in the perception of authentic leadership of the coach could have effects on the well-being of the coach. A study by Weiss et al. (2018) concluded that that mediated by a reduction in mental exhaustion, authentic leadership lessens the stress of leaders and increases work commitment. In addition, the timing of the training program's administration coincided with the final phase of the season where coaches are subjected to greater pressure in relation to the achievement of objectives, it could be considered that the program could positively affect well-being from the coach. From the perspective of the positive effects that authentic leadership to athletes, Bandura and Kavussanu (2018) conclude in the need to find ways encouraging coaches to adopt authentic leadership. Therefore, it could be considered that a program like the one developed in this study it could be one of those paths.

Other studies in which a leadership skills training program was carried out have evidenced coaches' greater perception of their leadership style and the variables that make it up (Newin et al., 2008; Vella and Perlman, 2014). With regard to the results obtained concerning the coach's perception of the justice that he/she promotes within the team, the results were significant for the experimental group when taking before and after measurements into account; post-training values being statistically significant. In the control group, no change was observed in terms of the perception of justice being promoted by the coaches. As with other research, coaches felt more confident in their ability to convey the decisions they made (Newin et al., 2008). In different contexts a connection has been made with organizational justice promoted as an important factor in determining leadership style (Gillet et al., 2013). Specifically, authentic leadership has been associated with organizational justice within the business environment (García-Guiu et al., 2015). In the realm of sports, Jowett (2005, 2009) relates a style of leadership and justice promoted by coaches to foster closeness, which includes emotional ties between the coach and his athletes, could facilitate a greater degree of commitment by both parties.

The results obtained after the training program oriented toward authentic leadership meant that the coaches' self-perception of competence, were significant for the experimental group, considering whether the measurement was taken before or after, with higher values obtained after having participated in the training program. No variation, meanwhile, was found for the control group and these results coincide with previous research (Conde et al., 2010; Soriano et al., 2014). When analyzing the subscales into which the questionnaire was divided, it was discovered that the interaction period of measurement^*^ group was significant for the subscale “teaching and instructing skills.” For the remaining subscales: “competence to motivate,” “competence to make decisions,” and “capacity to influence attitude,” the interaction period of measurement/group was not significant. The post-training increase occurring solely in the aforementioned subscale (“teaching and instructing skills”) may be due to the implementation of the training program on the part of the coaches, and the orientation of these acquisitions toward more procedural aspects that are related to their background (Mesquita et al., 2011b). In this sense, it seems that their assessment of the program has resulted in a stronger appreciation of their own competence in relation to procedural and technical issues. In line with other studies that administered a program with comparable objectives (Smith et al., 2007; Newin et al., 2008), the coaches gained greater confidence with respect to their own competence.

The results obtained for the coaches' self-efficacy were statistically significant for the experimental group when taking into account whether the data was coming from the first or second measurement period. In the control group, no significant modifications were detected regarding perceived self-efficacy between the two sets of data. We believe that the authentic leadership training program modulated the results obtained by the coaches with respect to the perception of their overall self-efficacy. On the other hand, it stands to reason that coaches' perception of self-efficacy may be influenced by their level of training, academic education, or coaching experience (Mesquita et al., 2011a). In this regard, we believe that the program may have generated an increase in coaches' perceived ability to solve problems within their domain. Bandura (2001) emphasizes that the convictions people use to achieve success better predict the perception of their self-efficacy beliefs. In this line, it stands to reason that coaches with higher levels of overall self-efficacy develop more favorable beliefs about their ability to solve problems. In view of the findings, we believe that the contents developed in the authentic training program could have positively influenced the enhanced self-efficacy of the coach. In our work, and also in other studies with similar characteristics (Lee et al., 2002), the training program increased perceived level of general self-efficacy.

The training program oriented toward authentic leadership in terms of the collective efficacy that coaches perceived within their teams indicated a significant increase among coaches who received the authentic leadership program. Collective efficacy is one of the greatest predictors of performance (Feltz and Lirgg, 1998), and in this sense, other studies have linked task-centered and less ego-driven behaviors to higher collective efficacy environments (Damato et al., 2011). In other words, the substantial shift in the values obtained with regard to collective efficacy leads us to believe that the authentic training program implemented alters the coaches' priorities by concentrating their efforts more closely on the task rather than the result. The perception of high collective efficacy is positively related to other relevant variables in team sports performance.

### Players

The results obtained with respect to the players' perception of their coach's authentic leadership were not significant for the experimental group. In the control group, a statistical trend was observed which marked a significant decrease in the players' perception of their coach's authentic leadership. It is striking to find the same result among the players in the control group as among their coaches. Both perceived a decrease in the authentic leadership that was developed. It is possible that the incidents that took place within the teams between the two data collection sessions, specifically in this final stretch of the season where relationships tend to deteriorate (García-Calvo et al., 2014b; González-Ponce, 2018), may have generated these results.

Regarding the level of competence that players perceived in their coaches, the results show a significant decrease in the perception of competence in the control group, both in the overall competence, and in two of the subscales of the questionnaire (competence to motivate and competence to influence attitude). For the experimental group, on the other hand, it was found that after the authentic leadership program the players felt that their coaches were more competent in making decisions. It was observed that the authentic training program not only generated an increase in the coaches' perception of self-competence, but that the players under their charge also identified an increase in the coaches' competence (in the subscale decision-making competence). Other studies that administered a leadership program have also found that players reported an improvement in their perception of their coach's competence after he or she had undergone training (Smith et al., 2007; Newin et al., 2008; Urra, 2018). The results in the two groups are therefore noteworthy, given that while the players in the experimental group perceived an increase in the decision-making competence of their coaches, the players in the control group showed a significant decrease in the perception of their coach's competence when he or she had not undergone training.

In terms of perceived justice as reported by the players, the results obtained show no difference between the two periods of measurement in either group. While the coaches in the experimental group perceive themselves to exhibit higher levels of justice, their players do not report the same perception. Regarding collective efficacy perceived by players, the results obtained did not show differences in either group, although other studies in which coaches were trained (Voight and Callaghan, 2001) did in fact reveal an increase in the efficacy perceived by athletes on collective performance.

To conclude, both hypotheses were partially confirmed. In addition, the authentic sports leadership training program had a remarkable effect not only on the coaches, but, for the different variables studied, that effect extended to their players as well.

These findings may have important practical implications. First, this study could be used to guide the organisms in charge of coach training to include leadership as a key aspect. Second, the results of this research should be taken into account by the head coaches of team sports. Likewise, sports clubs and associations could invest more in training programs aimed at developing authentic leadership among coaches as the positive effects involve not only the coaches but also their players. At last, authentic leadership training programs could help coaches and their assistant teams identify possible areas for improvement in their training and development as sports technicians.

### Limitations of the Study and Future Research

The sample size regarding coaches was small due to its having been selected from the National Degree of Higher Sports Technicians Schools with the highest level of training in both sports modalities. This helped to make a good selection of reputable coaches. A low power analysis sensitivity was found, but it did not impede that all the interactions time of measure^*^group showed significant, except for three subdimensions of the competence scale but not for the total score. Also, there was not a follow-up evaluation of the intervention. Future studies should address whether these results hold 6 months, or a year, after the conclusion of the intervention. Finally, the evaluation of the program was carried out exclusively through the application of questionnaires. Future studies of a qualitative nature could study the reasons why this program has been effective. It will also relevant to test the contents and number of sessions necessary for an effective intervention.

## Conclusions

The coach's self-perception of authentic leadership, perceived justice, competence, self-efficacy, and collective efficacy is favorably influenced by the training program. The players also perceive their coach as more competent after the training course. A 15-h intervention program was quite effective with such a short duration. This study highlights the benefit of authentic leadership training in sports coaches.

## Data Availability Statement

The original contributions presented in the study are included in the article/supplementary material, further inquiries can be directed to the corresponding author/s.

## Ethics Statement

Ethical approval was not provided for this study on human participants because it fulfilled all the precepts of the Declaration of Helsinki. The participants and their tutors were informed. Written informed consent to participate in this study was provided by the participants' legal guardian/next of kin.

## Author Contributions

DS: conceptualization, methodology, investigation, resources, data curation, writing—original draft, supervision, and project administration. JG: conceptualization, methodology, software, investigation, resources, writing—original draft, and project administration. RC: methodology, software, formal analysis, writing—original draft, and visualization. MS: investigation, writing—review and editing, and visualization. All authors contributed to the article and approved the submitted version.

## Conflict of Interest

The authors declare that the research was conducted in the absence of any commercial or financial relationships that could be construed as a potential conflict of interest.
